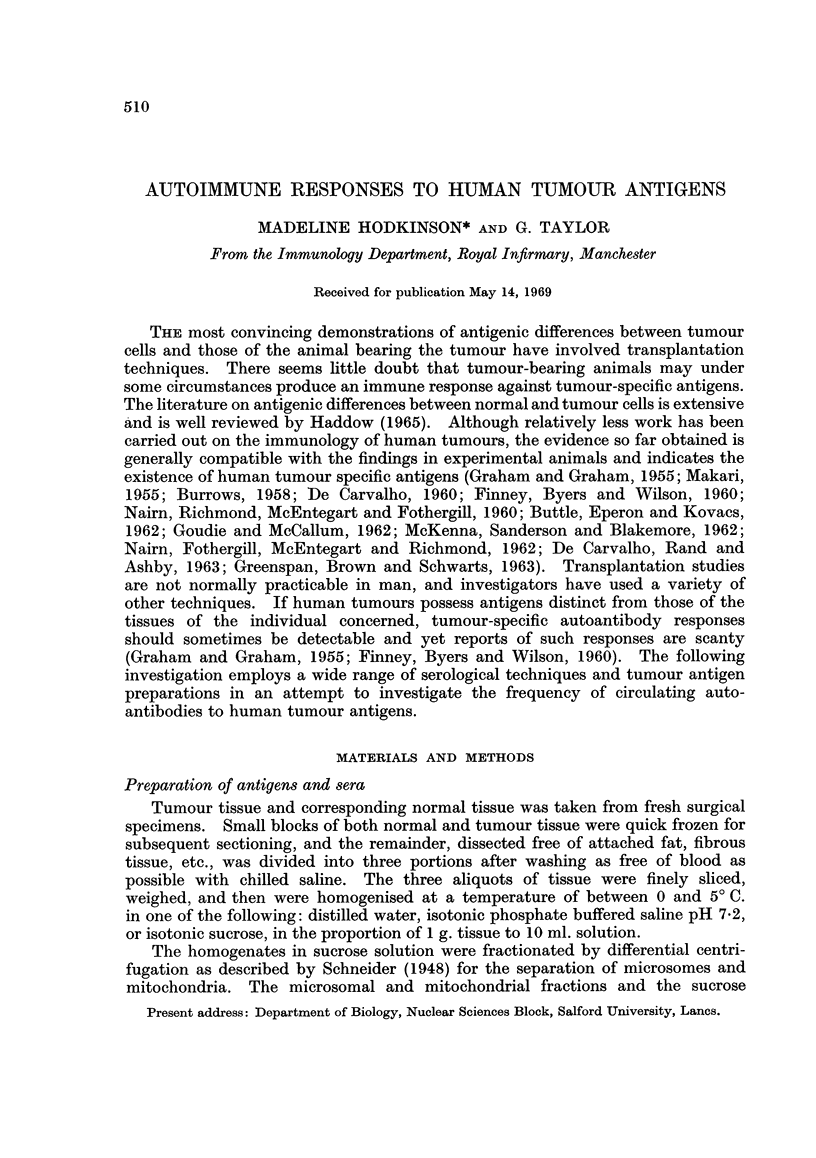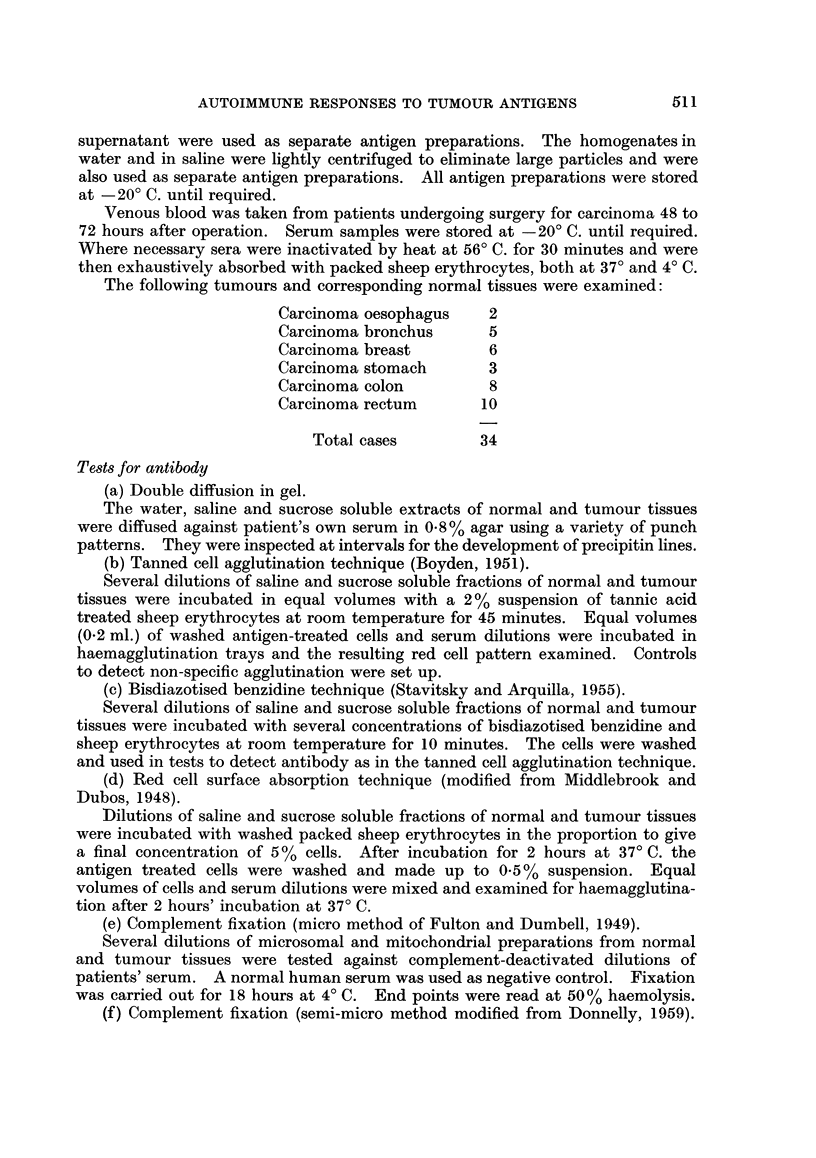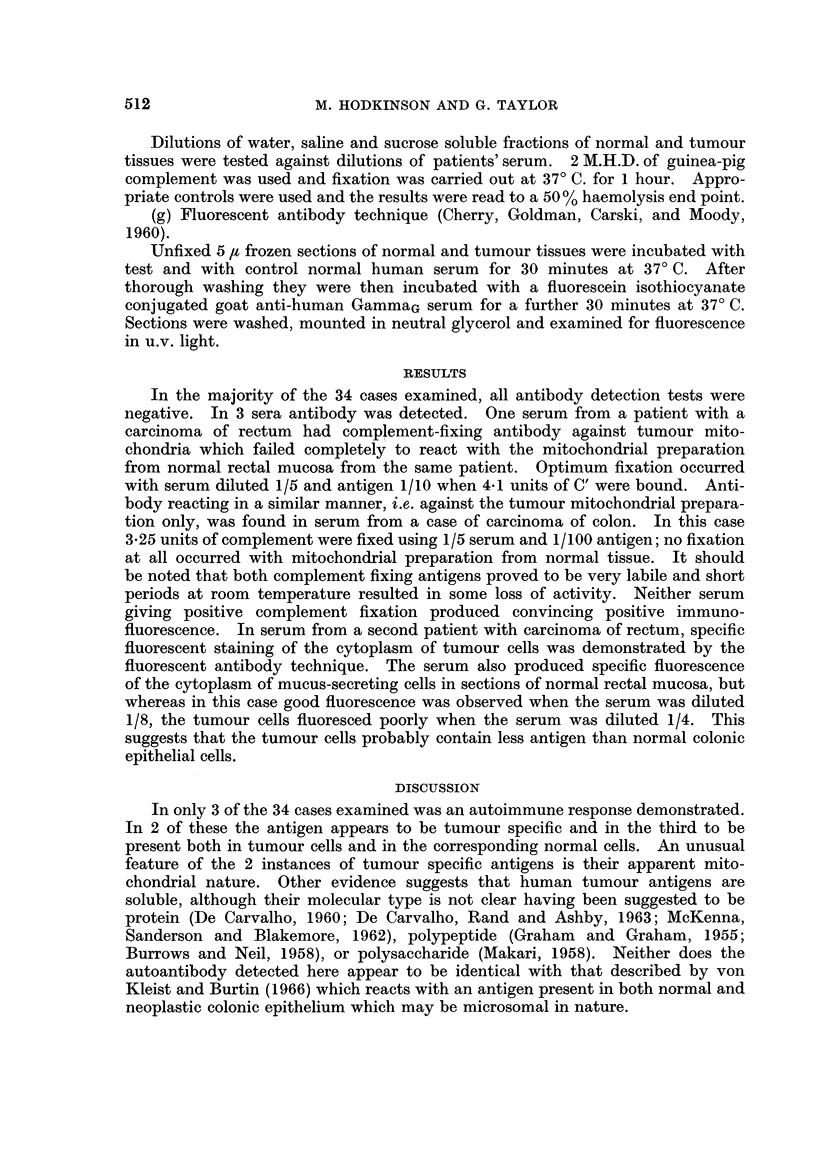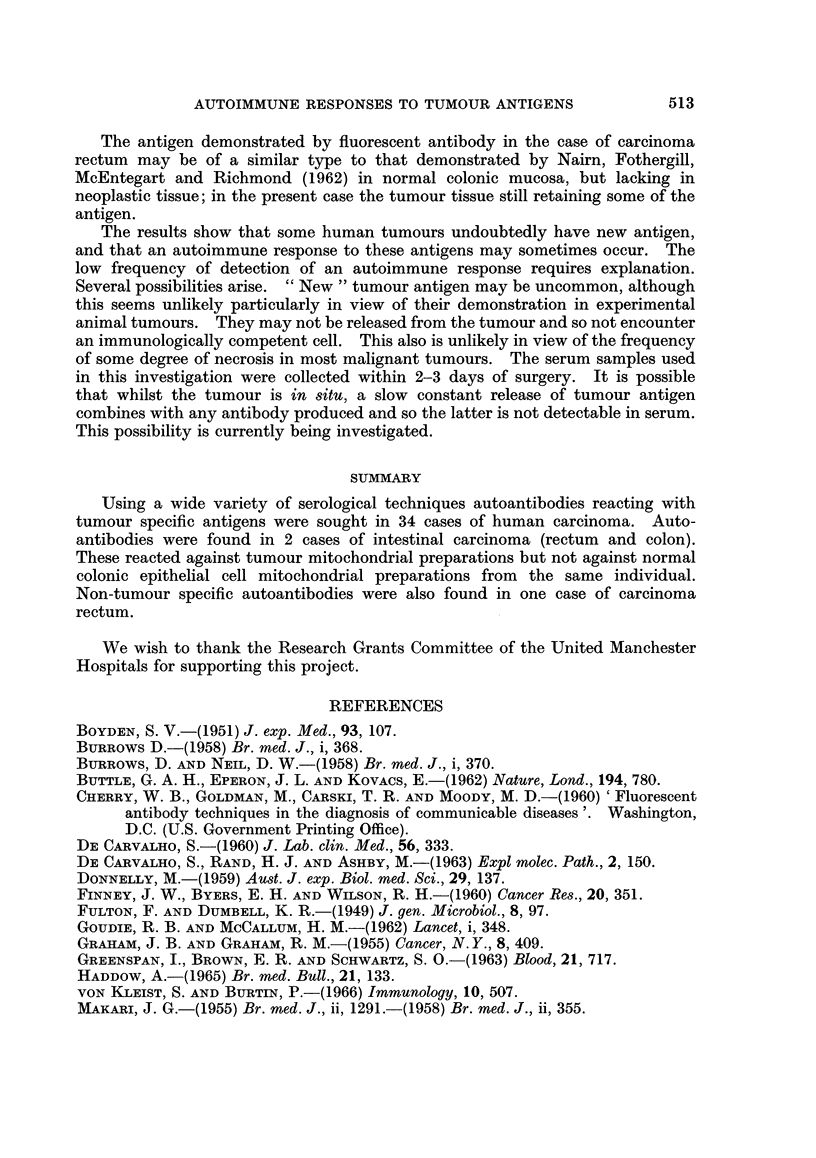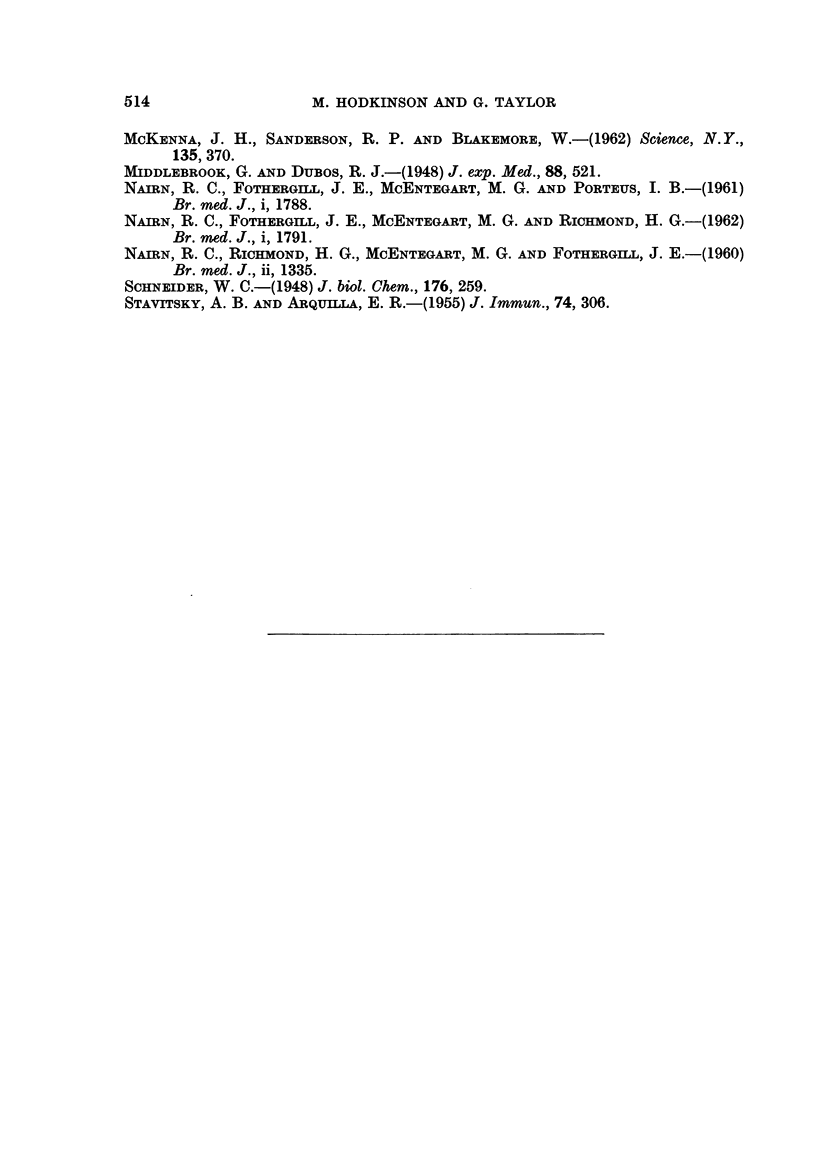# Autoimmune responses to human tumour antigens.

**DOI:** 10.1038/bjc.1969.63

**Published:** 1969-09

**Authors:** M. Hodkinson, G. Taylor


### AUTOIMMUNE RESPONSES TO HUMAN TUMOUR ANTIGENS

MADELINE HODKINSON* AND G. TAYLOR

*From the Immunology Department, Royal Infirmary, Manchester*

Received for publication May 14, 1969

THE most convincing demonstrations of antigenic differences between tumour cells and those of the animal bearing the tumour have involved transplantation techniques. There seems little doubt that tumour-bearing animals may under some circumstances produce an immune response against tumour-specific antigens. The literature on antigenic differences between normal and tumour cells is extensive and is well reviewed by Haddow (1965). Although relatively less work has been carried out on the immunology of human tumours, the evidence so far obtained is generally compatible with the findings in experimental animals and indicates the existence of human tumour specific antigens (Graham and Graham, 1955; Makari, 1955; Burrows, 1958; De Carvalho, 1960; Finney, Byers and Wilson, 1960; Nairn, Richmond, McEntegart and Fothergill, 1960; Buttle, Eperon and Kovacs, 1962; Goudie and McCallum, 1962; McKenna, Sanderson and Blakemore, 1962; Nairn, Fothergill, McEntegart and Richmond, 1962; De Carvalho, Rand and Ashby, 1963; Greenspan, Brown and Schwarts, 1963). Transplantation studies are not normally practicable in man, and investigators have used a variety of other techniques. If human tumours possess antigens distinct from those of the tissues of the individual concerned, tumour-specific autoantibody responses should sometimes be detectable and yet reports of such responses are scanty (Graham and Graham, 1955; Finney, Byers and Wilson, 1960). The following investigation employs a wide range of serological techniques and tumour antigen preparations in an attempt to investigate the frequency of circulating autoantibodies to human tumour antigens.

### MATERIALS AND METHODS

### *Preparation of antigens and sera*

Tumour tissue and corresponding normal tissue was taken from fresh surgical specimens. Small blocks of both normal and tumour tissue were quick frozen for subsequent sectioning, and the remainder, dissected free of attached fat, fibrous tissue, etc., was divided into three portions after washing as free of blood as possible with chilled saline. The three aliquots of tissue were finely sliced, weighed, and then were homogenised at a temperature of between 0 and 5° C. in one of the following: distilled water, isotonic phosphate buffered saline pH 7·2, or isotonic sucrose, in the proportion of 1 g. tissue to 10 ml. solution.

The homogenates in sucrose solution were fractionated by differential centrifugation as described by Schneider (1948) for the separation of microsomes and mitochondria. The microsomal and mitochondrial fractions and the sucrose

Present address: Department of Biology, Nuclear Sciences Block, Salford University, Lancs.

supernatant were used as separate antigen preparations. The homogenates in water and in saline were lightly centrifuged to eliminate large particles and were also used as separate antigen preparations. All antigen preparations were stored at −20° C. until required.

Venous blood was taken from patients undergoing surgery for carcinoma 48 to 72 hours after operation. Serum samples were stored at −20° C. until required. Where necessary sera were inactivated by heat at 56° C. for 30 minutes and were then exhaustively absorbed with packed sheep erythrocytes, both at 37° and 4° C.

The following tumours and corresponding normal tissues were examined:

| | |
|---|---|
| Carcinoma oesophagus | 2 |
| Carcinoma bronchus | 5 |
| Carcinoma breast | 6 |
| Carcinoma stomach | 3 |
| Carcinoma colon | 8 |
| Carcinoma rectum | 10 |
| Total cases | 34 |

*Tests for antibody*

(a) Double diffusion in gel.

The water, saline and sucrose soluble extracts of normal and tumour tissues were diffused against patient's own serum in 0·8% agar using a variety of punch patterns. They were inspected at intervals for the development of precipitin lines.

(b) Tanned cell agglutination technique (Boyden, 1951).

Several dilutions of saline and sucrose soluble fractions of normal and tumour tissues were incubated in equal volumes with a 2% suspension of tannic acid treated sheep erythrocytes at room temperature for 45 minutes. Equal volumes (0·2 ml.) of washed antigen-treated cells and serum dilutions were incubated in haemagglutination trays and the resulting red cell pattern examined. Controls to detect non-specific agglutination were set up.

(c) Bisdiazotised benzidine technique (Stavitsky and Arquilla, 1955).

Several dilutions of saline and sucrose soluble fractions of normal and tumour tissues were incubated with several concentrations of bisdiazotised benzidine and sheep erythrocytes at room temperature for 10 minutes. The cells were washed and used in tests to detect antibody as in the tanned cell agglutination technique.

(d) Red cell surface absorption technique (modified from Middlebrook and Dubos, 1948).

Dilutions of saline and sucrose soluble fractions of normal and tumour tissues were incubated with washed packed sheep erythrocytes in the proportion to give a final concentration of 5% cells. After incubation for 2 hours at 37° C. the antigen treated cells were washed and made up to 0·5% suspension. Equal volumes of cells and serum dilutions were mixed and examined for haemagglutination after 2 hours' incubation at 37° C.

(e) Complement fixation (micro method of Fulton and Dumbell, 1949).

Several dilutions of microsomal and mitochondrial preparations from normal and tumour tissues were tested against complement-deactivated dilutions of patients' serum. A normal human serum was used as negative control. Fixation was carried out for 18 hours at 4° C. End points were read at 50% haemolysis.

(f) Complement fixation (semi-micro method modified from Donnelly, 1959).

Dilutions of water, saline and sucrose soluble fractions of normal and tumour tissues were tested against dilutions of patients' serum. 2 M.H.D. of guinea-pig complement was used and fixation was carried out at 37° C. for 1 hour. Appropriate controls were used and the results were read to a 50% haemolysis end point.

(g) Fluorescent antibody technique (Cherry, Goldman, Carski, and Moody, 1960).

Unfixed 5 $\mu$ frozen sections of normal and tumour tissues were incubated with test and with control normal human serum for 30 minutes at 37° C. After thorough washing they were then incubated with a fluorescein isothiocyanate conjugated goat anti-human $Gamma_G$ serum for a further 30 minutes at 37° C. Sections were washed, mounted in neutral glycerol and examined for fluorescence in u.v. light.

### RESULTS

In the majority of the 34 cases examined, all antibody detection tests were negative. In 3 sera antibody was detected. One serum from a patient with a carcinoma of rectum had complement-fixing antibody against tumour mitochondria which failed completely to react with the mitochondrial preparation from normal rectal mucosa from the same patient. Optimum fixation occurred with serum diluted 1/5 and antigen 1/10 when 4·1 units of C′ were bound. Antibody reacting in a similar manner, *i.e.* against the tumour mitochondrial preparation only, was found in serum from a case of carcinoma of colon. In this case 3·25 units of complement were fixed using 1/5 serum and 1/100 antigen; no fixation at all occurred with mitochondrial preparation from normal tissue. It should be noted that both complement fixing antigens proved to be very labile and short periods at room temperature resulted in some loss of activity. Neither serum giving positive complement fixation produced convincing positive immunofluorescence. In serum from a second patient with carcinoma of rectum, specific fluorescent staining of the cytoplasm of tumour cells was demonstrated by the fluorescent antibody technique. The serum also produced specific fluorescence of the cytoplasm of mucus-secreting cells in sections of normal rectal mucosa, but whereas in this case good fluorescence was observed when the serum was diluted 1/8, the tumour cells fluoresced poorly when the serum was diluted 1/4. This suggests that the tumour cells probably contain less antigen than normal colonic epithelial cells.

### DISCUSSION

In only 3 of the 34 cases examined was an autoimmune response demonstrated. In 2 of these the antigen appears to be tumour specific and in the third to be present both in tumour cells and in the corresponding normal cells. An unusual feature of the 2 instances of tumour specific antigens is their apparent mitochondrial nature. Other evidence suggests that human tumour antigens are soluble, although their molecular type is not clear having been suggested to be protein (De Carvalho, 1960; De Carvalho, Rand and Ashby, 1963; McKenna, Sanderson and Blakemore, 1962), polypeptide (Graham and Graham, 1955; Burrows and Neil, 1958), or polysaccharide (Makari, 1958). Neither does the autoantibody detected here appear to be identical with that described by von Kleist and Burtin (1966) which reacts with an antigen present in both normal and neoplastic colonic epithelium which may be microsomal in nature.

The antigen demonstrated by fluorescent antibody in the case of carcinoma rectum may be of a similar type to that demonstrated by Nairn, Fothergill, McEntegart and Richmond (1962) in normal colonic mucosa, but lacking in neoplastic tissue; in the present case the tumour tissue still retaining some of the antigen.

The results show that some human tumours undoubtedly have new antigen, and that an autoimmune response to these antigens may sometimes occur. The low frequency of detection of an autoimmune response requires explanation. Several possibilities arise. " New " tumour antigen may be uncommon, although this seems unlikely particularly in view of their demonstration in experimental animal tumours. They may not be released from the tumour and so not encounter an immunologically competent cell. This also is unlikely in view of the frequency of some degree of necrosis in most malignant tumours. The serum samples used in this investigation were collected within 2–3 days of surgery. It is possible that whilst the tumour is *in situ*, a slow constant release of tumour antigen combines with any antibody produced and so the latter is not detectable in serum. This possibility is currently being investigated.

### SUMMARY

Using a wide variety of serological techniques autoantibodies reacting with tumour specific antigens were sought in 34 cases of human carcinoma. Autoantibodies were found in 2 cases of intestinal carcinoma (rectum and colon). These reacted against tumour mitochondrial preparations but not against normal colonic epithelial cell mitochondrial preparations from the same individual. Non-tumour specific autoantibodies were also found in one case of carcinoma rectum.

We wish to thank the Research Grants Committee of the United Manchester Hospitals for supporting this project.

### REFERENCES

Boyden, S. V.—(1951) *J. exp. Med.*, **93**, 107.
Burrows D.—(1958) *Br. med. J.*, i, 368.
Burrows, D. and Neil, D. W.—(1958) *Br. med. J.*, i, 370.
Buttle, G. A. H., Eperon, J. L. and Kovacs, E.—(1962) *Nature, Lond.*, **194**, 780.
Cherry, W. B., Goldman, M., Carski, T. R. and Moody, M. D.—(1960) ' Fluorescent antibody techniques in the diagnosis of communicable diseases '. Washington, D.C. (U.S. Government Printing Office).
De Carvalho, S.—(1960) *J. Lab. clin. Med.*, **56**, 333.
De Carvalho, S., Rand, H. J. and Ashby, M.—(1963) *Expl molec. Path.*, **2**, 150.
Donnelly, M.—(1959) *Aust. J. exp. Biol. med. Sci.*, **29**, 137.
Finney, J. W., Byers, E. H. and Wilson, R. H.—(1960) *Cancer Res.*, **20**, 351.
Fulton, F. and Dumbell, K. R.—(1949) *J. gen. Microbiol.*, **8**, 97.
Goudie, R. B. and McCallum, H. M.—(1962) *Lancet*, i, 348.
Graham, J. B. and Graham, R. M.—(1955) *Cancer, N.Y.*, **8**, 409.
Greenspan, I., Brown, E. R. and Schwartz, S. O.—(1963) *Blood*, **21**, 717.
Haddow, A.—(1965) *Br. med. Bull.*, **21**, 133.
von Kleist, S. and Burtin, P.—(1966) *Immunology*, **10**, 507.
Makari, J. G.—(1955) *Br. med. J.*, ii, 1291.—(1958) *Br. med. J.*, ii, 355.

McKenna, J. H., Sanderson, R. P. and Blakemore, W.—(1962) *Science, N.Y.*, **135**, 370.
Middlebrook, G. and Dubos, R. J.—(1948) *J. exp. Med.*, **88**, 521.
Nairn, R. C., Fothergill, J. E., McEntegart, M. G. and Porteus, I. B.—(1961) *Br. med. J.*, i, 1788.
Nairn, R. C., Fothergill, J. E., McEntegart, M. G. and Richmond, H. G.—(1962) *Br. med. J.*, i, 1791.
Nairn, R. C., Richmond, H. G., McEntegart, M. G. and Fothergill, J. E.—(1960) *Br. med. J.*, ii, 1335.
Schneider, W. C.—(1948) *J. biol. Chem.*, **176**, 259.
Stavitsky, A. B. and Arquilla, E. R.—(1955) *J. Immun.*, **74**, 306.